# An integrated insight into the response of bacterial communities to anthropogenic contaminants in a river: A case study of the Wonderfonteinspruit catchment area, South Africa

**DOI:** 10.1371/journal.pone.0216758

**Published:** 2019-05-21

**Authors:** K. Jordaan, A. M. Comeau, D. P. Khasa, C. C. Bezuidenhout

**Affiliations:** 1 Unit for Environmental Sciences and Management, Microbiology, North-West University, South Africa, Potchefstroom, South Africa; 2 Institut de Biologie Intégrative et des Systèmes (IBIS), Université Laval, Québec, Canada; USDA-ARS Salinity Laboratory, UNITED STATES

## Abstract

Bacterial communities in human-impacted rivers and streams are exposed to multiple anthropogenic contaminants, which can eventually lead to biodiversity loss and function. The Wonderfonteinspruit catchment area is impacted by operational and abandoned gold mines, farms, and formal and informal settlements. In this study, we used 16S rRNA gene high-throughput sequencing to characterize bacterial communities in the lower Wonderfonteinspruit and their response to various contaminant sources. The results showed that composition and structure of bacterial communities differed significantly (*P*<0.05) between less (downstream) and more (upstream) polluted sites. The taxonomic and functional gene dissimilarities significantly correlated with each other, while downstream sites had more distinct functional genes. The relative abundance of *Proteobacteria*, *Bacteroidetes* and *Actinobacteria* was higher at upstream sites, while *Acidobacteria*, *Cyanobacteria*, *Firmicutes* and *Verrucomicrobia* were prominent at downstream sites. In addition, upstream sites were rich in genera pathogenic and/or potentially pathogenic to humans. Multivariate and correlation analyses suggest that bacterial diversity was significantly (*P*<0.05) impacted by pH and heavy metals (cobalt, arsenic, chromium, nickel and uranium). A significant fraction (~14%) of the compositional variation was explained by a combination of anthropogenic inputs, of which mining (~6%) was the main contributor to bacterial community variation. Network analysis indicated that bacterial communities had non-random inter- and intra-phyla associations and that the main taxa showed both positive and negative linkages to environmental parameters. Our results suggest that species sorting, due to environmental parameters, was the main process that structured bacterial communities. Furthermore, upstream sites had higher relative abundances of genes involved in xenobiotic degradation, suggesting stronger removal of polycyclic aromatic hydrocarbons and other organic compounds. This study provides insights into the influences of anthropogenic land use on bacterial community structure and functions in the lower Wonderfonteinspruit.

## Introduction

Increasing anthropogenic disturbances (e.g., mining, urban and rural settlements, sewage works, and agriculture) on freshwater systems accelerate deterioration of water quality and ecosystem health. Given these detrimental effects, there is an urgent need to address nonpoint source water pollution (NPS) and to assess the state of such water sources for both immediate and future uses.

Bacterial communities in freshwaters play key roles in biogeochemical cycles. They are responsible for breaking down organic material and remineralizing nutrients, which in turn affect energy flux and circulation of material in the system [1, 2]. Bacterial diversity and species abundance are in turn associated with nutrient availability and the physical environment [3, 4]. Changes in nutrient sources and the environment may have major repercussions on community composition and species abundance, affecting overall water quality [5, 6]. Determining which chemical and physical factors correlate with community changes will reveal how microorganisms react to different perturbations and increase our understanding of microbial ecology and their effects on pollution [7, 8]. By combining this approach with animal and plant ecology, specialists may be able to develop an effective remediation strategy for polluted waters [9].

The recent development of high-throughput sequencing technologies, taxonomic reference databases and bioinformatics tools provide great opportunities to explore bacterial communities in aquatic ecosystems and their responses to perturbations [10, 11]. However, relatively little is known about the associations between contaminants and the resident microbiome, nor the latter’s role in the functioning and service of such systems [12, 13]. Examining co-occurrence patterns has the potential to identify ecological processes that structure bacterial communities and how communities respond to perturbations [14, 15]. Patterns of inter-taxa associations may reveal the niche spaces shared by community members, or even more direct symbioses between community members given that closely related taxa may share ecological traits and life styles [14, 16].

The lower Wonderfonteinspruit (WFS) River receives high contamination loads from nearby gold mines, domestic wastewater treatment plants (WWTPs), urban and informal settlements, and agricultural runoff [17, 18]. Due to excessive pollution, including microorganisms, the water quality of the river has degraded markedly and continues to deteriorate. Polluted stream water and/or mine effluent (adjacent canals) are often used by communities for domestic purposes, or watering of livestock and commercial crops [19]. Extensive research has covered the effects of gold mining on the water quality of the WFS and its tributaries, in particular uranium [18, 20]. However, little data exist on the bacterial diversity and community composition (BCC) in the WFS in relation to the above-mentioned anthropogenic inputs. In this context, the aim of the study was to assess the impacts of anthropogenic disturbances on the BCC in the lower WFS. Water samples were collected along the longitudinal profile of the river that reflected point and non-point sources of water pollution. Bacterial communities were analysed with 16S rRNA gene amplicon pyrosequencing and correlated with environmental parameters to achieve the following goals: (i) to evaluate how bacterial communities respond to different pollution inputs; (ii) to determine the driving forces in shaping the bacterial community distributions; and (iii) to explore co-occurrence patterns in bacterial communities along the river.

## Methods and methods

### Study sites

**Fig 1:**
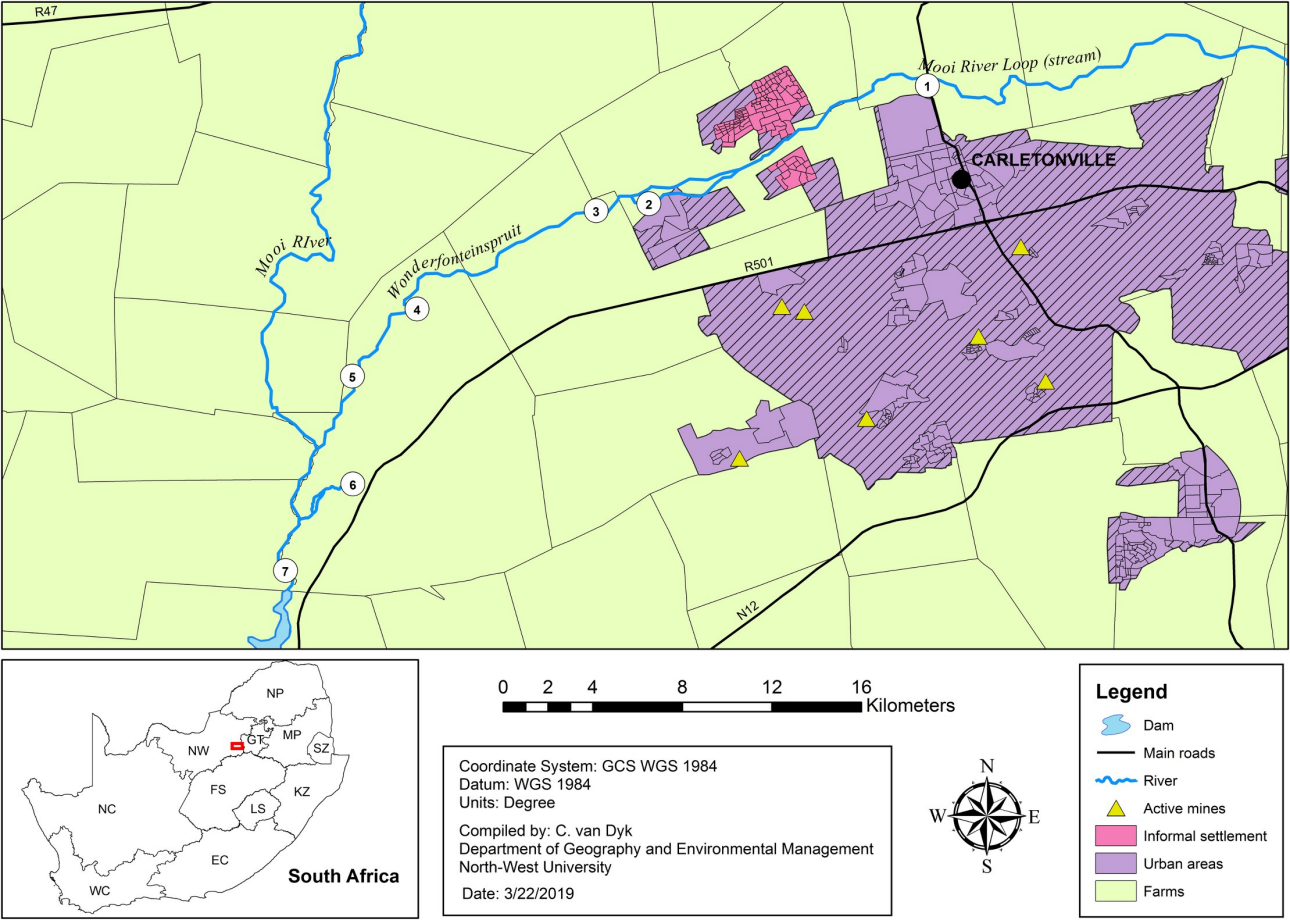
Geographical map of the lower Wonderfonteinspruit. Illustrated is the general location of the study site in the North West Province, with a detailed view of the sampling sites examined for bacterial community composition.

The Wonderfonteinspruit Catchment Area (WCA) originates in the southern part of Krugersdorp on the Witwatersrand ridge (Gauteng Province) (Fig 1). From there the river flows in a south-easterly direction through municipal and mining areas before confluence with the Mooi River upstream of the city of Potchefstroom (North West Province) [17, 18]. The upper section of the WCA is situated in the Gauteng Province and the lower part of the catchment is in the North West Province [17].

This study was conducted in the lower WCA in spring and summer of 2012 (October—December). Samples were collected from seven sites to represent a wide range of water quality data and assess the effects of anthropogenic activities on the water resource (Fig 1; S1 Table). Study sites included: Site 1—Carletonville area (gold mining activities, formal and informal settlements), 26°18'57.0"S 27°22'56.9"E; Site 2—Welverdiend (formal settlement), 26°22'01.9"S 27°16'14.1"E; Site 3—Department of Water Affairs and Forestry (DWAF) monitoring point C2H069 (downstream of Welverdiend and all major discharge points from gold mines in the area), 26°22'12.1"S 27°14'57.8"E; Site 4—karst spring from the Turffontein dolomitic eye and farming community, 26°24'34.2"S 27°10'38.7"E; Site 5—Muiskraal (farming community), 26°26'11.3"S 27°09'05.1"E; Site 6—karst spring from the Gerhard Minnebron dolomitic eye and farming community, 26°28'47.3"S 27°09'05.8"E; Site 7—point downstream of the confluence with the Mooi River, 26°30'52.4"S 27°07'28.3"E. No specific sampling permissions were required for locations accessible to the general public (site 1–3 & 7), while permission was granted by private land owners to conduct sampling on site (site 4–6). Field studies did not involve endangered or protected species.

### Sample collection

Freshwater samples were collected in autoclaved sterilized 2 L glass containers and placed at 4°C in the dark until filtration, normally within 8 h after collection. Samples were taken in duplicate from each sampling site to determine bacterial community composition, chemical water quality, and heavy metals. Physico-chemical parameters measured *in situ* included temperature, pH and electrical conductivity (EC). Selected chemical and heavy metal elements were analysed by Eco-Analytica Laboratory, South Africa. Chemical parameters included chloride (Cl^-^), nitrate (NO_3_^-^), phosphate (PO_4_^3^^-^), sulphate (SO_4_^2^^-^), and bicarbonate (HCO_3_^-^). Trace metals measured included manganese (Mn), iron (Fe), cobalt (Co), nickel (Ni), copper (Cu), chromium (Cr), zinc (Zn), selenium (Se), lead (Pb), cadmium (Cd), mercury (Hg), arsenic (As), and uranium (U).

### DNA isolation and PCR amplification

Total DNA from water samples was isolated immediately following filtration as described previously [21]. Briefly, DNA was isolated from 0.2 μm nitrocellulose membrane filters by homogenization and enzymatic cell lysis using a lysozyme (1 mg/mL) and proteinase K (1 mg/mL) solution. Following lysis, total genomic DNA was captured and purified from the crude lysate on silica membranes in a spin column format (PeqGold Bacterial DNA Kit; PEQLAB Biotechnologie GmbH, Erlangen, Germany). The quality and quantity of the isolated nucleic acids were determined using the Nanodrop ND1000 (NanoDrop Technologies, Wilmington, DE, USA) and agarose electrophoresis [21]. The isolated DNA was stored at –20°C until further analysis.

The V6—V8 region of the 16S rRNA gene was amplified using the bacterial-specific primer pair reported elsewhere [22]. PCR reactions (50 μL) contained: 5μL Q5 reaction buffer (New England BioLabs Inc., Ipswich, MA, USA), 0.2 mM of each dNTP, 0.2 μM of each primer, 1 U of Q5 High-Fidelity DNA polymerase (New England BioLabs), PCR-grade water, and 1–3 μL of template DNA. Three separate DNA dilutions were used for each sample: 1, 0.5 and 0.1X (concentrations ranged between 0.5 and 52 ng). Cycling conditions were as follows: initial denaturation at 98°C for 30 s; 30 cycles of denaturation at 98°C for 10 s, annealing at 55°C for 30 s, and extension at 72°C for 30 s; followed by a final extension at 72°C for 2 min. Triplicate reactions for each sample were pooled and purified using Agencourt AMPure beads (Beckman Coulter Inc., Brea, CA, USA). The quality of pooled samples was evaluated using the Agilent DNA 7500 Chip Kit (Agilent Technologies Inc., Santa Clara, CA, USA) and Agilent 2100 Bioanalyzer (Agilent Technologies Inc.).

### 454-pyrosequencing

To characterise BCC along the lower WFS, pyrosequencing of the above PCR products was performed at the IBIS/Université Laval Plate-forme d’Analyses Génomiques (Québec, Canada) using the Roche 454 GS-FLX Titanium chemistry. Data analysis was conducted with mothur v.1.39.5 [23]. Raw sequence data was quality trimmed and checked for chimeras, the singletons were removed. Final high-quality sequences were assigned to operational taxonomic units (OTUs) at 97% identity and rarefaction curves were constructed to determine whether sampling depth was sufficient to accurately characterize the BCC. Taxonomic classification was based upon the Greengenes reference database [24] with a 97% bootstrap confidence threshold.

DNA sequences were submitted to the GenBank database as BioProject PRJNA275052.

### Statistical analysis

All statistical analyses were performed with R software version 3.3.3 (http://www.r-project.org). One-way analysis of variance (ANOVA), with Tukey’s *post hoc* test (*P*<0.05), was performed on environmental parameters to determine significant differences of the parameters by sampling location.

Statistical analyses on community data were performed using rarefied (3774 sequences per sample), log_10_(x+1)-normalized data unless otherwise indicated. A range of diversity indices were calculated using the vegan package. Specifically, we estimated richness (i.e. observed OTUs), Shannon index, Inverse Simpson index and Pielou’s evenness as measures of alpha-diversity. Beta-diversity analyses involved clustering of samples using the Bray-Curtis distance metric, while overlap in membership between communities was estimated using the Jaccard index. The resulting distance matrices were visualized using non-metric multidimensional scaling (NMDS) with ggplot2. Analysis of molecular variance (AMOVA, [25]) was applied in mothur to test whether the spatial separation of the sampling locations visualized in the NMDS plots was statistically significant. Calculations were based on both Bray-Curtis and Jaccard distance metrics, 1000 permutations and a false discovery rate (FDR) of 0.05. To examine changes in beta-diversity, we performed homogeneity of the group variances using beta-dispersivity tests (PERMDISP) and permutational multivariate analysis of variance (PERMANOVA) with the functions *betadisper* and *adonis* (vegan package), respectively. Tukey’s HSD (honest significant difference) was used as a *post hoc* test to determine which of the groups (upstream vs. downstream) and/or sites differed in variance.

Forward selection of Hellinger transformed community data and standardized environmental parameters were performed, using function *ordistep* (vegan package), to find the set of parameters that could best explain the variation in BCC. Distance-based RDA (db-RDA; *capscale* function in the vegan package) was then used to evaluate the effects of environmental parameters on community composition. Spearman-rank correlations were used to identify correlations between individual taxa and environmental parameters. Correction for multiple-testing of the *P*-values was performed according to the Benjamini-Hochberg method [26]. The respective contributions of anthropogenic activities (i.e. mining, urban, informal settlements, agriculture, bacterial pathogens and heavy metals) and environmental variables to microbial community variations were further evaluated with variance partitioning analysis (*varpart* function in the vegan package; [27]). To avoid redundancy and multicollinearity in variation partitioning, the importance of factors and variables were first tested with RDA using forward selection and 1000 permutations. Only variables with a VIF<5 and *P*<0.05 were selected for further analysis. For each data set of variables, forward selection was performed separately including anthropogenic inputs (anthropogenic source, type of pollution and potential pathogens), heavy metals, and environmental parameters. All heavy metal and environmental data were respectively log_10_-normalized and z-score standardized prior to analysis.

Microbial metabolic pathways were estimated based on the 16S rRNA gene data using PICRUSt [28]. The OTU data set generated in mothur was used to prepare biom files formatted as input for PICRUSt v.1.1.0 [28] with the *make*.*biom* script available in mothur. OTU abundances were mapped against the Greengenes (v13.8) database at a 97% identity level. The rarefied OTU table was used to normalize the 16S rRNA gene copy number and KEGG Orthologs (KOs; [29, 30]) were predicted from the normalized data. Predicted metagenomes were then inferred to KEGG Pathways and gene counts were normalized to relative abundance using predicted functional trait settings. The accuracy of metagenome predictions was estimated using the Nearest Sequenced Taxon Index (NSTI) scores [28]. A pairwise statistical comparison of the relative metabolic functions between upstream and downstream sites was carried out using STAMP [31], two sided G-test (w/Yates’) + Fisher’s statistical test with the DP: asymptotic-CC confidence interval method with the Benjamini-Hochberg FDR multiple test correction using a *P*-value of <0.05 [32]. To simplify analysis any non-microbial categories, for example ‘Human Diseases’, were excluded from further analysis. Mantel tests were performed to assess correlations between functional and taxonomic community dissimilarity matrices based on Bray-Curtis distance and visualized in NMDS plots. PERMANOVA was used to test whether upstream and downstream bacterial communities harbour significantly different metagenomes.

Co-occurrence networks were generated comprising of consistently-detected and highly-abundant OTUs (i.e., the “core” community): the community data were filtered by using only those OTUs with a relative abundance >0.2%. This filtering step removed poorly represented OTUs and reduced the network complexity [14], resulting in a core community of 385 OTUs.

Non-random co-occurrence patterns of OTUs were first tested with the checkerboard score (C-score) under the null hypothesis of random community assembly [33], where 50,000 matrices were randomly generated from the filtered community data (function *oecosimu* with *nestedchecker* and *quasiswap* in the bipartite package). Standardized effect size (SES) was used as a measure of OTU segregation as previously described [34].

Using the filtered OTU data set (absolute abundances) and z-score-standardized environmental parameters, all possible Spearman correlations and corresponding *P* values were calculated. Correction for multiple-testing of the *P*-values was performed according to the Benjamini-Hochberg method [26]. Spearman correlations were sorted for statistical significance (*P*<0.05) with coefficient (ρ) ≥ ±0.6 [35]. Significant relationships were then selected and translated into networks in Cytoscape 3.5.1 [36]. The topological properties of the network were subsequently analysed with the NetworkAnalyzer tool [37]. Modular structure and groups of highly interconnected nodes were identified using the MCODE application [38] with standard parameters. Taxa with the highest degree (>10) and betweenness centrality values (>0.02) were considered as keystone taxa [39].

## Results

### Physico-chemical analysis

Physico-chemical parameters and trace metals are summarized in Tables 1 and 2. Amongst the seven sampling locations, site 1 had significantly (*P*<0.05) higher values for temperature, pH, SO_4_^2^^-^, NO_3_^-^, Cl^-^, Co, Cu and As than those at other sites. No significant differences were found for the remainder of the environmental parameters at the sampling locations. Sulphate levels for the December samples increased considerably (>200 mg/L) and exceeded the target water quality range (TWQR) for domestic use, although the water is not directly used for domestic purposes (S2 Table) [40]. Bicarbonate reached maximum and minimum levels in November and December, respectively. November was associated with exceptionally hot and dry weather that could have caused accumulation of bicarbonate levels in the river, while December experienced heavy rainfall and flushed a large quantity of bicarbonate ions. Nitrate concentrations at sites 1, 4, 5 and 6 were at all times above the TWQR for domestic use. Although EC remained relatively constant throughout the sampling period, concentrations of dissolved salts were above the TWQR for domestic use. Heavy metals were consistently higher at the upstream sites (sites 1–3), but were within the TWQR for domestic use, irrigation and livestock watering, with the exception of Fe. Iron levels were at all times above the recommended TWQR for domestic use and reached a maximum concentration of 0.68 mg/L.

**Table 1:** Physico-chemical variables measured in the lower Wonderfonteinspruit.

	Temp _(°C)_	pH	EC _(mS/m)_	PO_4_^3^‾ _(mg/L)_	SO_4_^2^‾ _(mg/L)_	NO_3_^‾^ _(mg/L)_	Cl^‾^ _(mg/L)_	HCO_3_^‾^ _(mg/L)_
Site1	23.07 ± 3.66	8.29 ± 0.07	91.50 ± 1.14	25.40 ± 40.70	220.46 ± 95.27	10.44 ± 0.98	59.16 ± 7.26	91.88 ± 108.92
Site2	21.47 ± 4.80	7.90 ± 0.21	88.50 ± 4.12	1.37 ± 0.22	193.26 ± 71.93	2.60 ± 2.63	61.89 ± 6.92	95.49 ± 116.01
Site3	20.83 ± 3.67	7.76 ± 0.04	95.13 ± 2.39	0.84 ± 0.43	225.22 ± 109.29	2.17 ± 1.72	73.50 ± 9.40	98.67 ± 118.92
Site4	20.20 ± 0.44	7.17 ± 0.03	75.93 ± 0.06	0.03 ± 0.04	100.17 ± 42.74	12.04 ± 0.95	39.54 ± 4.13	115.30 ± 155.31
Site5	20.30 ± 2.25	7.80 ± 0.25	77.90 ± 0.85	0.32 ± 0.54	119.34 ± 51.20	7.25 ± 1.70	44.71 ± 2.93	113.71 ± 151.60
Site6	21.23 ± 0.40	7.33 ± 0.03	77.17 ± 0.49	0.01 ± 0.0	113.40 ± 30.61	9.72 ± 5.63	44.75 ± 7.56	99.15 ± 127.43
Site7	21.30 ± 1.56	7.80 ± 0.07	71.50 ± 3.63	0.01 ± 0.0	111.66 ± 72.60	7.13 ± 4.43	39.20 ± 10.72	115.43 ± 160.17

Values are given as the mean of three replicates ± standard deviation

**Table 2:** Heavy metals concentrations measured in the lower Wonderfonteinspruit.

	Al _(ug/L)_	As _(ug/L)_	Cd _(ug/L)_	Co _(ug/L)_	Cr _(ug/L)_	Cu _(ug/L)_	Fe _(ug/L)_	Hg _(ug/L)_	Ni _(ug/L)_	Mn _(ug/L)_	Pb _(ug/L)_	Se _(ug/L)_	U _(ug/L)_	Zn _(ug/L)_
Site1	20.00 ± 20.00	9.30 ± 1.50	0.05 ± 0.07	14.40 ± 3.15	0.13 ± 0.10	10.70 ± 5.46	430.00 ± 100.00	0.40 ± 0.66	50.00 ± 13.70	30.00 ± 40.00	8.05 ± 0.33	0.57 ± 0.82	37.00 ± 3.05	1.39 ± 1.75
Site2	180.00 ± 260.00	3.64 ± 1.39	0.05 ± 0.07	7.03 ± 1.12	0.60 ± 0.75	12.60 ± 5.87	480.00 ± 190.00	0.50 ± 0.48	9.55 ± 3.27	50.00 ± 80.00	8.18 ± 0.26	0.66 ± 0.94	19.90 ± 4.84	1.74 ± 2.36
Site3	20.00 ± 30.00	2.05 ± 2.06	0.05 ± 0.06	5.74 ± 1.73	0.69 ± 0.90	9.66 ± 2.37	400.00 ± 60.00	0.12 ± 0.18	19.50 ± 17.80	10.00 ± 10.00	8.48 ± 1.09	0.64 ± 0.90	17.00 ± 12.20	1.28 ± 1.54
Site4	0.32 ± 0.34	0.22 ± 0.31	0.05 ± 0.07	3.45 ± 0.43	0.77 ± 1.06	6.19 ± 1.81	370.00 ± 80.00	0.07 ± 0.10	2.28 ± 3.88	1.29 ± 2.22	8.14 ± 0.28	0.68 ± 0.96	3.20 ± 0.18	1.30 ± 1.53
Site5	7.29 ± 7.37	0.20 ± 0.28	0.05 ± 0.06	3.32 ± 0.25	0.44 ± 0.48	8.34 ± 3.37	390.00 ± 60.00	0.06 ± 0.062	2.50 ± 2.22	8.76 ± 13.50	8.14 ± 0.52	0.66 ± 0.94	18.80 ± 1.55	1.80 ± 2.37
Site6	2.55 ± 4.25	0.22 ± 0.31	0.06 ± 0.07	6.77 ± 1.91	1.29 ± 1.95	8.34 ± 6.43	360.00 ± 60.00	0.03 ± 0.03	0.06 ± 0.05	0.93 ± 1.58	8.19 ± 0.45	0.61 ± 0.84	4.58 ± 2.66	1.72 ± 2.22
Site7	20.00 ± 30.00	0.22 ± 0.30	0.05 ± 0.06	5.58 ± 1.94	0.43 ± 0.47	5.92 ± 2.50	350.00 ± 70.00	0.01 ± 0.01	0.35 ± 0.55	6.66 ± 11.20	8.16 ± 0.20	0.68 ± 0.97	5.76 ± 2.15	1.34 ± 1.53

Values are given as the mean of three replicates ± standard deviation

### Bacterial community composition across sampling locations

A total of 157,073 reads were obtained from the seven sampling sites. Following quality filtering and processing, 30,764 reads were used for further analysis. Reads for site 4 (October) were removed from the total data set before equalising and re-merging the bar-coded files. The number of quality trimmed sequences for this site in October was markedly low and therefore the sample was omitted to prevent a major loss of data which might have given an inaccurate representation of the BCC. The quality filtered and chimera-free reads from the sample libraries were clustered into 5,969 OTUs at a 97% sequence identity cut-off using the average-neighbour clustering method for the entire dataset. None of the rarefaction curves reached saturation at a 97% identity level, indicating that the full extent of taxonomic diversity was not surveyed (S1 Fig).

**Fig 2:**
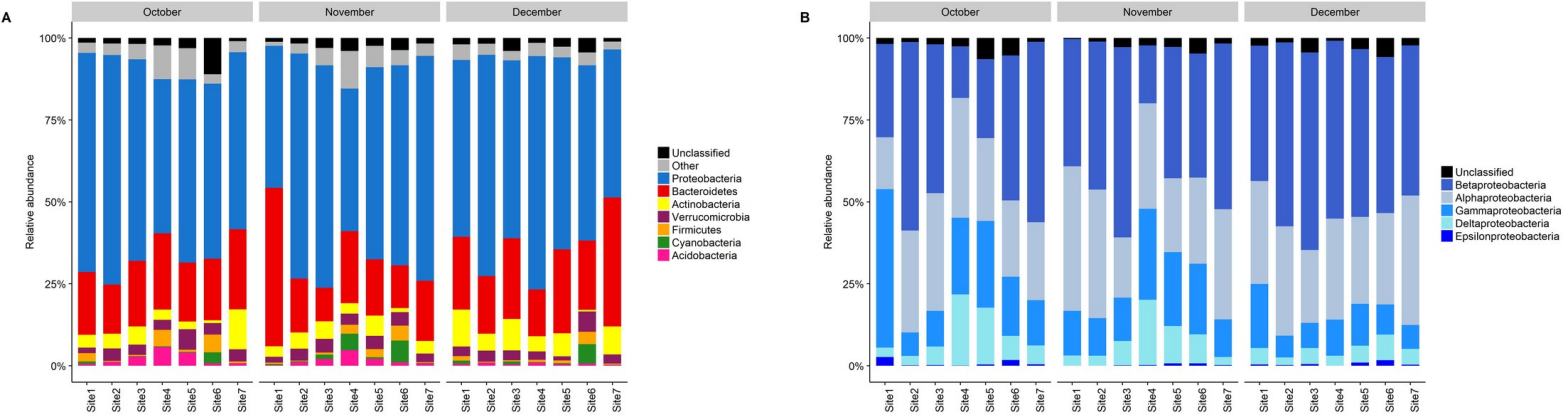
Taxonomic compositions of bacterial communities. Composition is based on all sequences detected at the different sampling locations at (A) phylum- and (B) Proteobacteria class level.

The bacterial communities at the seven sampling locations were primarily dominated by *Proteobacteria* (58.85±1.99%) followed by *Bacteroidetes* (21.26±1.97%), *Actinobacteria* (5.2±0.72%), *Verrucomicrobia* (3.4±0.28%), *Firmicutes* (1.44±0.35%), *Cyanobacteria* (1.35±0.47%) and *Acidobacteria* (1.33±0.28%) (Fig 2A). The *Proteobacteria* were distributed between sites (in order of abundance) as *Betaproteobacteria* (26.87±2.01%), *Alphaproteobacteria* (16.99±1.04%), *Gammaproteobacteria* (9.38±1.41%), *Deltaproteobacteria* (3.67±0.52%), and *Epsilonproteobacteria* (0.35±0.10%) (Fig 2B). The relative abundance at the phylum level varied across the different sampling locations (Fig 2A). Notable differences included higher relative abundances of *Acidobacteria*, *Cyanobacteria*, *Firmicutes* and *Verrucomicrobia* at the downstream sites (site 4–6), while *Bacteroidetes* was prominent at more polluted sites (site 1). The rare proportion of bacterial communities, which made up less than 1% of the total BCC, belonged to a diverse range of phyla including *Armatimonadetes*, AC1, BRC1, *Caldiserica*, *Caldithrix*, *Chlamydiae*, *Chlorobi*, *Chloroflexi*, *Elusimicrobia*, FBP, FCPU426, *Fibrobacteres*, *Fusobacteria*, GN02, GN04, GOUTA4, *Gemmatimonadetes*, H-178, KSB3, LD1, *Lentisphaerae*, NC10, NKB19, *Nitrospirae*, OC31, OD1, OP11, OP3, OP8, PAUC34f, *Planctomycetes*, SC4, *Spirochaetes*, TM6, TM7, TPD-58, *Tenericutes*, *Thermi*, WPS-2, WS1, WS3, WS4, WWE1 and ZB3 (Fig 2A). Seven OTUs of the classes *Alphaproteobacteria*, *Betaproteobacteria*, *Gammaproteobacteria* and *Flavobacteriia* (*Bacteroidetes*) were detected in all samples and were among the most abundant OTUs for each of the sampling locations. All of the seven OTUs were successfully classified at family level as *Rhodobacteraceae*, *Comamonadaceae*, *Aeromonadaceae* and *Flavobacteriaceae*.

Genera that represented >1% each of the total BCC included *Rhodobacter* (5.69±0.67%), *Flavobacterium* (3.91±1.04%), *Polynucleobacter* (2.37±0.43%), *Sediminibacterium* (2.15±0.3%), *Fluviicola* (1.46±0.32%), *Aeromonas* (1.37±0.70%), *Pseudomonas* (1.14±0.29%) and *Rhodoferax* (1.03±0.40%). As expected, several human opportunistic pathogens were identified throughout the stream, with site-to-site variations in relative abundances. Potential pathogens with moderate to high relative abundance (>0.4%) included *Flavobacterium*, *Sphingopyxis*, *Aeromonas*, *Pseudomonas*, *Achromobacter*, *Rhodoferax*, *Ralstonia* and *Acinetobacter*. Overall, site 1 had the highest abundance of potential pathogens during all three sampling periods followed by sites situated in farming communities.

### Alpha- and beta-diversity of bacterial communities

**Fig 3:**
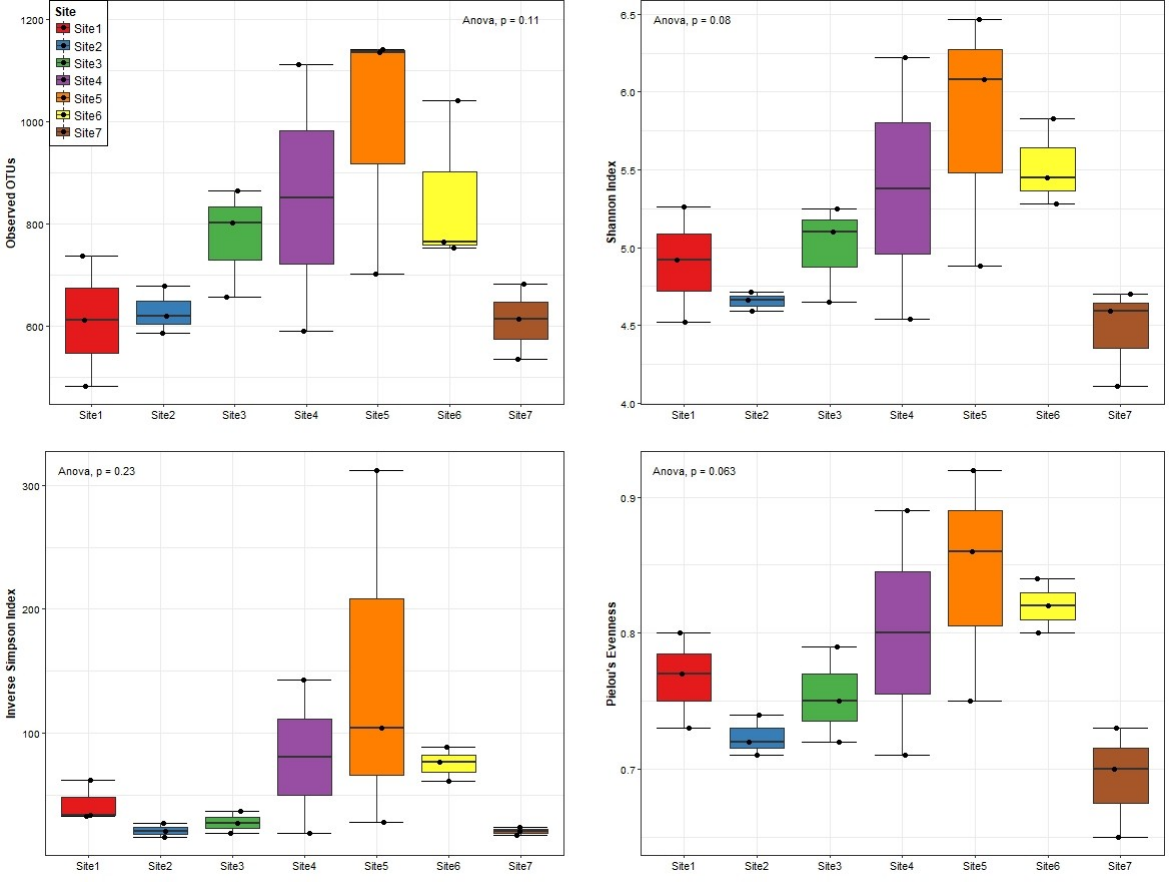
Alpha diversity indices grouped per sampling location. Analyses were performed using the 97% identity level OTU table rarefied to 3774 sequences per sample. The samples are grouped and coloured coded by location interval.

Alpha-diversity, calculated at the 97% identity level using a range of indices including richness (i.e. observed OTUs), Shannon- and Inverse Simpson indices, and Pielou’s evenness, are shown in Fig 3. Overall, alpha-diversity for the downstream sites (site 4–6) was richer, more diverse, and more even as compared to the upstream sites (site 1–3). Strikingly, lower richness, diversity and evenness were also observed for site 7 and may be attributed to the effects of wash-out dynamics on bacterial communities due to the confluence of the two water bodies. Consistently, the average number OTUs (992±145), Shannon index (5.8±0.5), Inverse Simpson index (148±85) and Pielou’s evenness (0.84±0.05) were highest for site 5. In contrast, site 7 exhibited the lowest average richness (611±42), diversity (Shannon: 4.5±0.2; Inverse Simpson: 21±2) and evenness (0.69±0.02). In light of the observed location-specific differences, we assessed whether the variation in alpha-diversity was significant within any given sampling location. The ANOVA results indicated significant differences (*P*<0.05) in bacterial diversity (Shannon index) and evenness between site 5 and 7, while no significant differences (*P*>0.05) were observed in bacterial community richness, diversity and evenness for the other sites.

**Fig 4:**
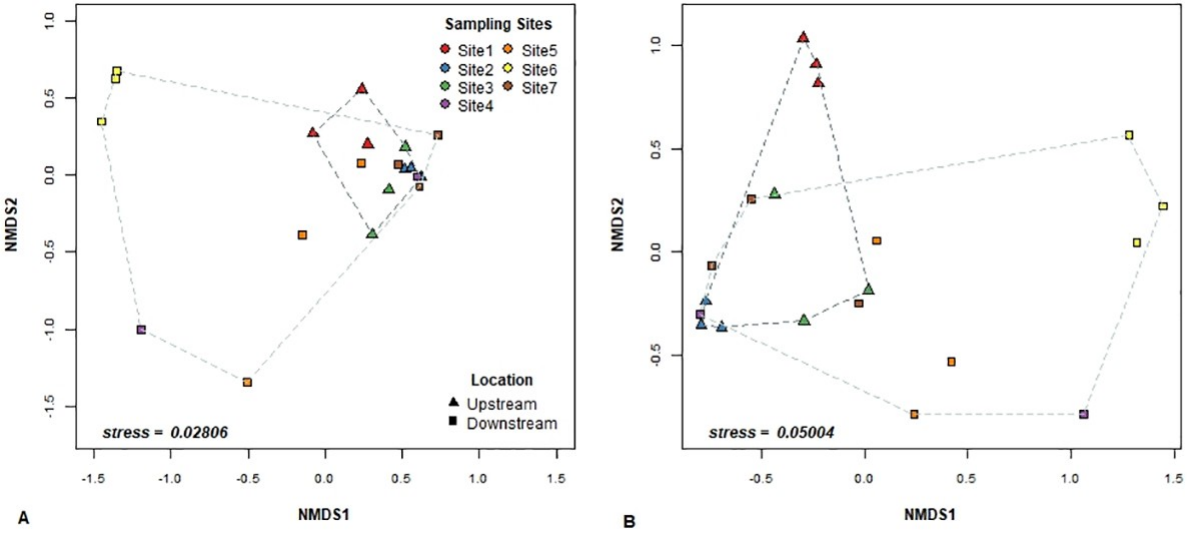
Non-metric Multidimensional Scaling (NMDS) plots of bacterial communities at all sampling locations. Plots were generated using (A) structure-based Bray-Curtis distance and (B) membership-based Jaccard distances.

Beta-diversity ordination plots (Fig 4) indicated that upstream and downstream sites had different taxonomic communities, with a stronger clustering observed for the Bray-Curtis metric (Fig 4A) as compared to the Jaccard metric (Fig 4B). The AMOVA and beta-dispersivity results showed significant differences between upstream and downstream locations and depended on the type of beta-diversity metric being used, i.e. community structure (Bray-Curtis) or community membership (Jaccard). Overall, the majority of changes in beta-diversity using AMOVA were observed for community membership (*p* = 0.02) rather than for community structure (*p* = 0.046). In the case of the PERMANOVA and beta-dispersivity test, our results suggest that the sampling locations (upstream vs. downstream) differed in dispersion when both community structure (PERMANOVA, F = 1.77, *p* = 0.045; PERMDISP, F = 5.24, *p* = 0.034) and community membership (PERMANOVA, F = 1.37, *p* = 0.008; PERMDISP, F = 6.46, *p* = 0.02) were considered.

### Associations between physico-chemical water characteristics, trace metals and BCC

**Fig 5:**
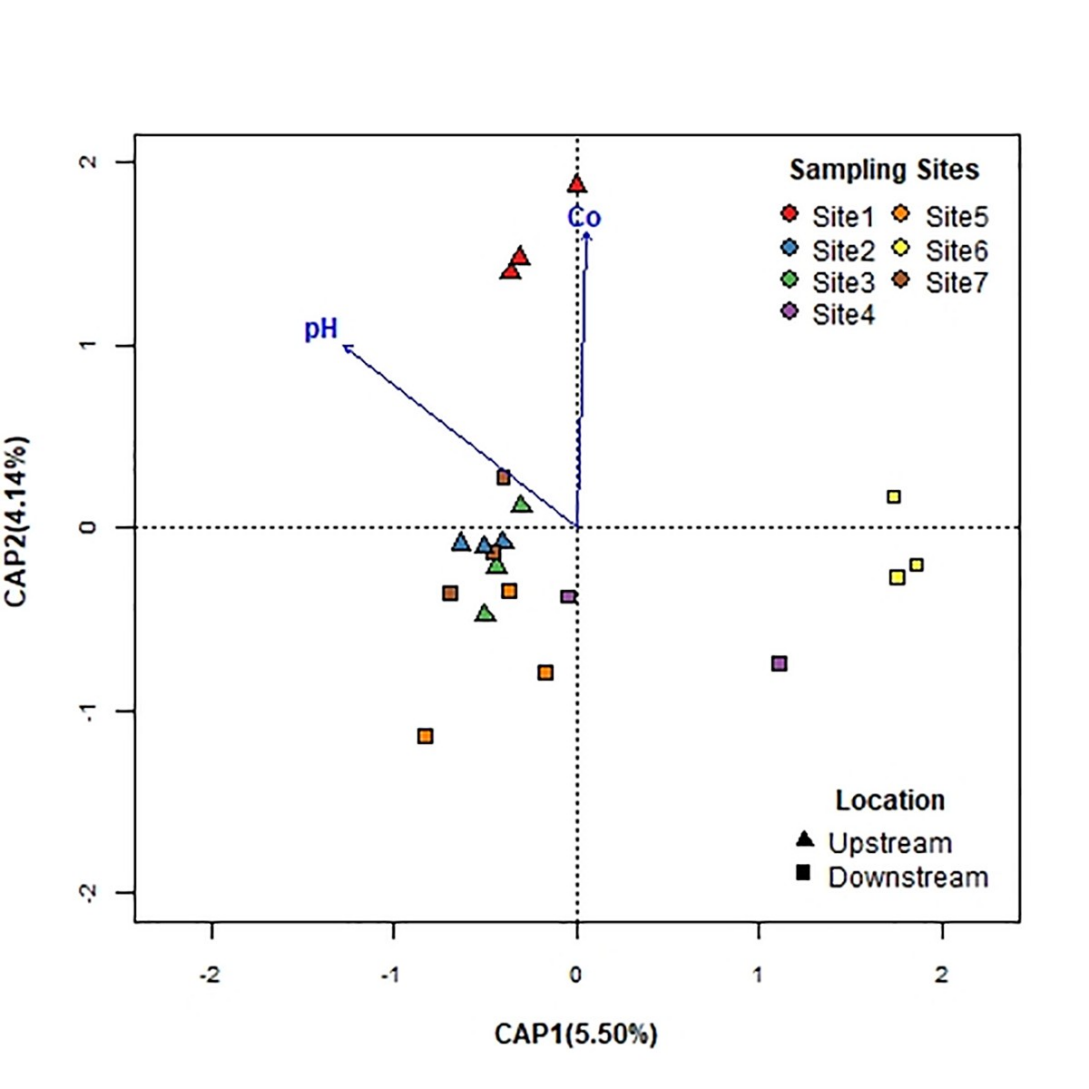
Distance-based redundancy analysis (db-RDA) bi-plot of bacterial communities at all sampling locations with environmental parameters. Only pH and cobalt (Co) significantly explained variability in bacterial community structures and are fitted to the ordination (blue arrows).

The distanced-based redundancy analysis with forward selection (db-RDA; Fig 5) revealed that pH (F = 1.88, *p* = 0.009), together with cobalt (F = 1.85, *p* = 0.007), had a significant influence on BCC. However, these parameters only accounted for 10% of the total variation, suggesting that other unmeasured factors were important in driving the community dynamics.

**Fig 6:**
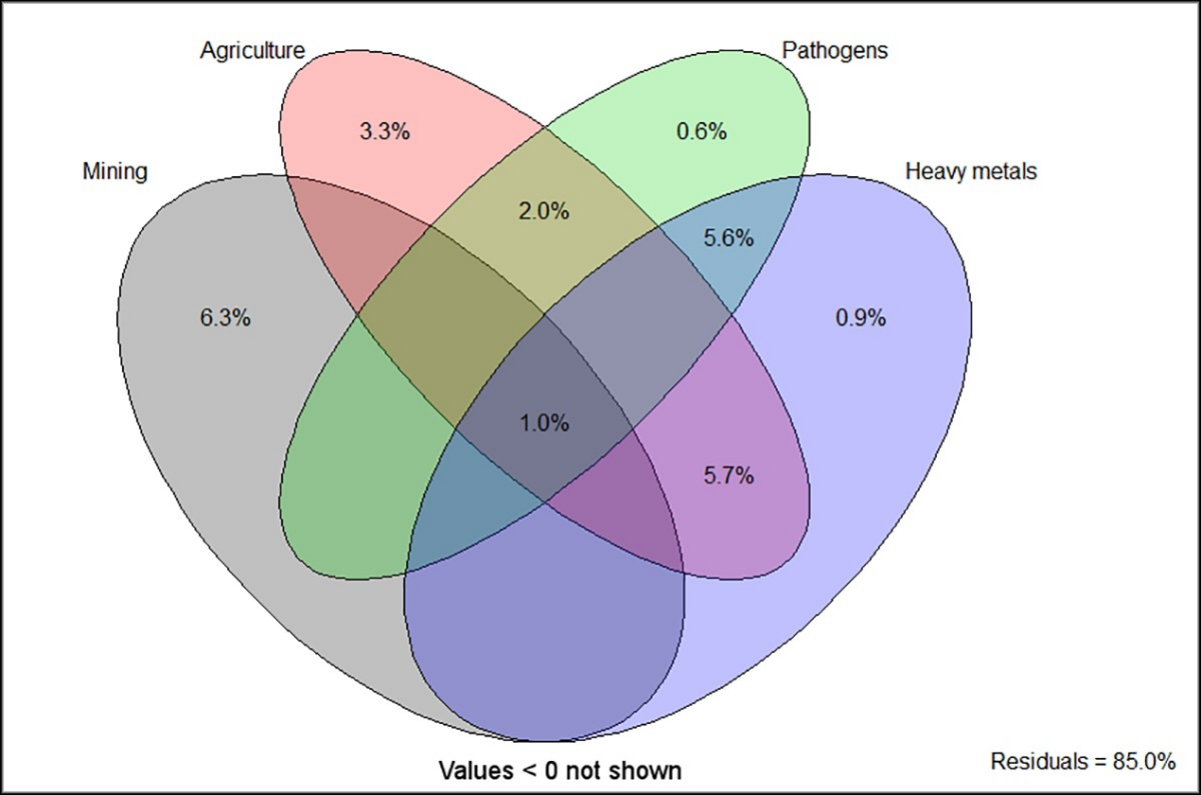
Variation partitioning results of BCC explained by anthropogenic inputs. Values indicate the percentage of variation explained (R^2^_adj_). A forward selection procedure was used to select the best variables in each data set important to BCC variation.

The combined effects of anthropogenic inputs (i.e. mining, agriculture, heavy metals and potential pathogens from sewage sources) on BCC significantly (*P*<0.05) explained a small portion of the variation in community composition (Fig 6). Together, all components explained 14.97% of BCC variation. Mining only accounted for ~6% of the variation, while agriculture, potential pathogens and heavy metals explained 3.3%, 0.6% and 0.9% of the variation, respectively. The interactions between the different anthropogenic inputs were moderate (2–5%), indicating that their effects were to a certain extent dependent on each other. More than 80% of the community variation could not be explained by the four components.

The extent of individual environmental variables on taxon distribution was also assessed by Spearman correlations with multiple test correction. *Alphaproteobacteria* (OTU-20, 129, 164, 313, 1508) had strong positive correlations with both pH and cobalt. In addition, pH was positively correlated with *Betaproteobacteria* (OTU-1329) and *Actinobacteria* (OTU-1266), while cobalt showed positive correlations with *Bacteroidetes* (OTU-416, 555), *Nitrospirae* (OTU-229) and *Gammaproteobacteria* (OTU-786). Interestingly, many of the physico-chemical and heavy metal parameters were positively correlated with several individual taxa although RDA suggested they were not key drivers in community dynamics. For example, sulphate, phosphate and nitrate were positively correlated with *Alphaproteobacteria* (OTU-210, 875, 1508, 874), *Betaproteobacteria* (OTU-131, 1329, 675) and *Bacteroidetes* (OTU-9, 312, 448, 271, 565, 948, 1384, 2200). Furthermore, these groups, together with *Gammaproteobacteria*, had strong correlations with arsenic, chromium, nickel and uranium. These findings suggest that the *Proteobacteria* and *Bacteroidetes* play a pivotal role in nutrient cycling and heavy metal resistance or absorption in the WFS.

### PICRUSt predicted metabolic characterization of upstream and downstream sites

**Fig 7:**
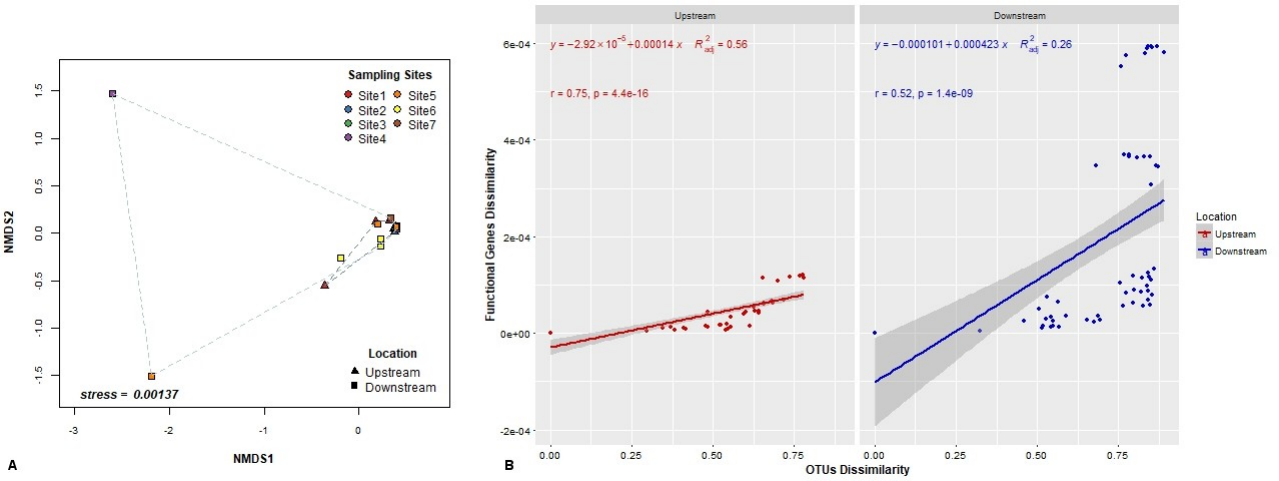
**Bray-Curtis dissimilarities of (A) the relative abundance of functional genes predicted by PICRUSt using NMDS analysis and (B) Mantel tests showing the relationship between the functional genes and OTU dissimilarities.** Red dots and line denote upstream sites. Blue dots and line denote downstream sites. Pearson correlation coefficient (r), statistical significance (*p*) and adjusted R-squared (R^2^_adj_) of linear regression are indicated. Grey lines indicate 95% confidence intervals.

The NSTI scores for all samples ranged between 0.187 and 0.188 with an overall mean of 0.19 ± 0.000125 s.d.; which is comparable to NSTI values reported for microbial communities in aquatic environments [32, 41]. Predicted metabolic functions did not differ significantly between upstream and downstream communities (PERMANOVA, F = 1.50, *p* = 0.31), although noticeable differences were evident between sites 1, 4 and 5 from NMDS analysis (Fig 7A). Mantel correlation tests revealed significantly positive correlations between the taxonomic and functional dissimilarities in upstream (R^2^ = 0.56, *p*<0.01) and downstream (R^2^ = 0.26, *p*<0.01) communities (Fig 7B). Moreover, the slope of the linear relationships was smaller in the upstream sites than in the downstream sites.

A total of 233 pathways (excluding human-related and/or poorly categorized) were indicated at the three-tier level of functional categories defined by the PICRUSt hierarchy. In general, upstream communities had higher relative abundances for genes associated with environmental information processing, while downstream assemblages showed higher relative abundances for genes essential to metabolism, cellular processes and signalling, and genetic information processing (S2A Fig). At the functional subcategory level (level two), upstream communities were enriched with genes involved in membrane transport, metabolism (glycolysis, fermentation, amino acid and lipid metabolism) and biodegradation of xenobiotics (S2B Fig). Genes involved in xenobiotics degradation included benzoate and aminobenzoate, caprolactam, naphthalene, ethylbenzene, polycyclic aromatic hydrocarbons, nitrotoluene and the degradation of terpenoids and polyketides. In contrast, predicted functional capacities for downstream communities included energy metabolism (oxidative phosphorylation and photosynthesis), cell motility and membrane structure, signal transduction, metabolism (enzymes, cofactors and vitamins, glycan biosynthesis, nucleotides), replication and repair of nucleotides, and folding, sorting and degradation of proteins. Higher resolution analysis of the predicted metabolic pathways showed that downstream assemblages have the potential to metabolize sulphur and nitrogen. Additionally, both upstream and downstream assemblages show potential to fix carbon in prokaryotes and/or photosynthetic organisms, although communities at upstream sites had a stronger carbon metabolism than in downstream sites.

### Bacterial co-occurrence patterns

Following the stringent conditions set for network construction (i.e. statistically significant (*P*<0.05) Spearman correlations with ρ ≥ ±0.6), 334 bacterial OTUs and 6 environmental parameters were used for subsequent analysis. Non-random co-occurrence patterns were detected by the C-score test using the filtered OTU data set. The observed C-score indicated that bacterial OTUs were not randomly distributed among the sampling locations (C-score = 12.80, *p*<0.001) and exhibited segregated patterns (SES = 75.67).

**Fig 8:**
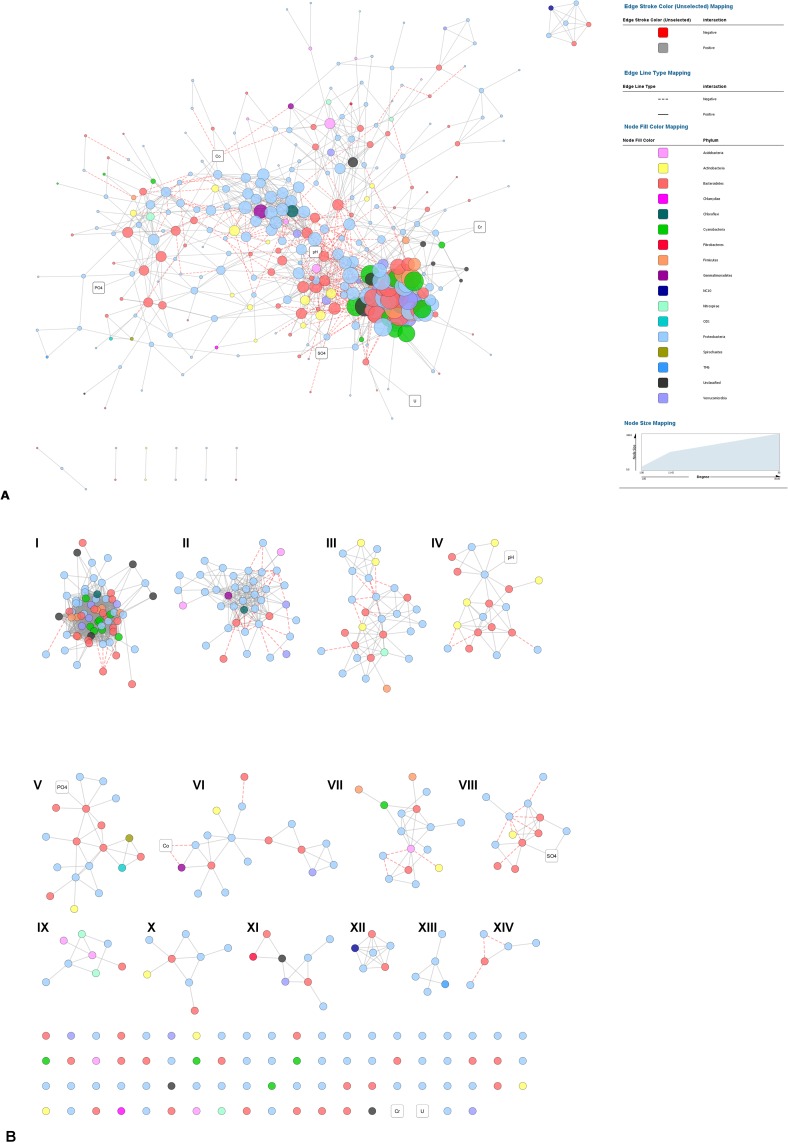
Network analysis of bacterial core OTUs (i.e. OTUs with relative abundance >0.2%) and environmental parameters. Co-occurrence patterns were based on significant (*P*<0.05) Spearman correlations with ρ ≥ ±0.6 showing either **(A)** the entire network or **(B)** modules of MCODE clustering of the complete network with default parameters. Modules are numbered from I-XIV. Each node (circle) in the network, representing a unique OTU, is coloured by phylum and the size is proportional to node degree. Each edge (connection) represents a strong and significant correlation (*P*<0.05), while the colour relates to the type of interaction: positive (grey solid lines) or negative (red dashed lines). Environmental parameters are presented by white rectangles.

The complete bacterial network (Fig 8A) consisted of 340 nodes and 1941 edges (positive = 1776, negative = 165). The average network distance between all pairs of nodes (characteristic path length) was 4.15 edges with a diameter of 10 edges. The network had a clustering coefficient of 0.39, a density value of 0.034 and a modularity index of 0.55. Overall, the network was comprised of highly connected nodes structured among modules and forming a highly clustered and modular topology.

The nodes in the network were assigned to 8 bacterial phyla and 17 classes. Among these, 5 taxa (*Alphaproteobacteria*, *Betaproteobacteria*, *Gammaproteobacteria*, *Deltaproteobacteria* and *Bacteroidetes*) were widely distributed, accounting for ~ 72% of all nodes. Based on centrality scores (betweenness centrality>0.02 and degree>10) the top taxa identified as keystone taxa included *Acidobacteria*, *Actinobacteria*, *Bacteroidetes*, *Cyanobacteria*, *Gemmatimonadetes* and *Proteobacteria* (*Alphaproteobacteria*, *Betaproteobacteria*, *Gammaproteobacteria* and *Deltaproteobacteria*). It is worth noting that many of the keystone taxa had a high relative abundance and did not show habitat preference. Instead, they were largely dispersed between sites (S3 Fig). As such, the majority of bacterial OTUs showed to be distributed throughout the river (S4 Fig). However, certain bacterial phylotypes were restricted to only upstream (e.g. *Lacibacter* spp.) or downstream (e.g. *Elstera* spp.) sites suggesting niche-based preferences. Although the network was dominated by edges between bacterial OTUs, connections between OTUs and environmental parameters were detected. For example, Co, pH and SO_4_^2^^-^ were amongst the most connected environmental nodes, illustrating their importance in the network. Other examples of strong linkages between OTUs and environmental variables include heavy metals (U and Cr) and PO_4_^3^^-^.

The MCODE algorithm generated 14 modules (I-XIV) of nodes which included OTUs belonging to numerous taxa and with varying abundances (Fig 8B). The primary module (module I) consisted of 68 nodes and 1006 edges, most of which were related to the downstream sites. Furthermore, we observed strong intra- and inter-phyla positive co-occurrence patterns (indicative of co-presence/mutualism) between keystone taxa within the primary module. Many of these associations were between: (i) phylotypes within the *Bacteroidetes* (e.g., *Paludibacter* with *Bacteroides*, *Flavobacterium* and *Runella*); (ii) *Cyanobacteria* and *Bacteroidetes* (e.g., *Phormidium* and *Runella*); (iii) *Firmicutes* and *Bacteroidetes* (e.g., *Pelosinus* with *Bacteroides*, *Flavobacterium* and *Runella*); and (iv) *Proteobacteria* (*Alpha*- and *Deltaproteobacteria*) with *Bacteroidetes* (e.g., *Magnetospirillum* and *Desulfovibrio* with *Runella*). Although the primary module consisted mainly of positive co-occurrences, several negative associations were also observed (indicative of mutually exclusive interactions). These were related to associations between *Bacteroidetes* with *Proteobacteria* (*Alphaproteobacteria* and *Betaproteobacteria*) and *Cyanobacteria*. Modules IV-VI and VIII structured around physico-chemical variables and heavy metals showed largely positive associations with keystone taxa, in particular *Bacteroidetes* (*Lacibacter* and *Cyclobacteriaceae* spp.), *Alphaproteobacteria* (*Sphingopyxis*), and *Betaproteobacteria* (*Oxalobacteraceae* spp.). Interestingly, cobalt was the only heavy metal that showed a negative co-occurrence with *Proteobacteria (Gammaproteobacteria)* and *Gemmatimonadetes*. For the remainder of the modules *Alpha*, *Beta*- and *Gammaproteobacteria* showed strong intra-phyla associations, the majority of which were positive, although negative co-occurrences also existed.

## Discussion

Our aim was to evaluate the impacts of anthropogenic activities on BCC in the lower WFS using a combination of approaches such as high-throughput sequencing and network analysis. The data set presented in this study is the first to taxonomically characterize bacterial communities in the WFS. As expected, pollutant levels varied along the longitudinal profile of the river and reflected point and non-point sources of water pollution. Many of the large active gold mines discharge fissure and process water into the WFS [17]. In addition, the stream receives discharge effluent from numerous point and diffuse sources such as old and/or abandoned mines, deposits of mining/milling slimes dams, wastewater treatment works, formal and informal settlements, peat mining, other industries and agriculture [18]. As a result, the water quality of the WFS and underlying dolomitic groundwater compartments have been substantially polluted by radionuclides, heavy metals, sulphates, organic constituents and biological material [42]. According to combined environmental and pyrosequencing data, site 1 had the greatest contamination loads. In general, the physico-chemical and heavy metal levels were significantly higher at this site as compared to the other sites. Furthermore, our results revealed high diversity and relative abundance of potential pathogens, many of which are associated with human stool, animal faeces (particularly swine and cattle) and domestic WWTP’s (e.g., *Corynebacterium*, *Microbacterium*, *Bacteroides*, *Clostridium*, *Aeromonas*, *Enterobacter*, *Enterococcus*). Lowest pollutant levels were found at sites located in farming communities, although the impacts of dry land agriculture were evident in the water quality. Our results appear to be well supported by the fact that gold mines in the “Far West Rand”, comprising of mines in Bekkersdal, Westonarea and Carletonville, contribute significantly to the pollution levels in the WFS [19, 43]. Moreover, sewage works at Carletonville and Khutsong (informal settlement) discharge sewage effluents to the WFS between Harry’s dam and the C2H069 monitoring point.

Bacterial diversity patterns in this study were similar to those previously observed in other 16S rRNA gene sequencing studies of freshwater ecosystems impacted by anthropogenic activities, in particular heavy metals [44–46]. These included a total of 52 phyla, but most samples were dominated by *Proteobacteria*, *Bacteroidetes*, *Actinobacteria*, *Verrucomicrobia*, *Firmicutes*, *Cyanobacteria* and *Acidobacteria*, with site-to-site variation in relative abundance. As expected, bacterial diversity was lower for the upstream sites, particularly site 1, where pollution was the greatest. Even though bacterial diversity in the WFS may be underestimated to a certain extent because rarefaction curves did not reach a plateau, it is still higher than what was obtained in streams impacted by anthropogenic activities [46, 47].To date, it is generally accepted that simple systems are vulnerable to perturbations, so bacterial communities require relatively high diversity, through spatial and temporal variability, to maintain their functions [48]. Also, previous studies have demonstrated that increased concentrations of organic and inorganic nutrients can stimulate bacterial growth resulting in higher bacterial diversity and community composition [49, 50]. However, our results do not support these findings, but comply with similar studies that demonstrated lower bacterial in heavily contaminated freshwater systems [44, 46, 51]. The lower bacterial diversity could be explained by the high abundance of generalists accompanied by the loss of the most sensitive species. Microbial communities often adapt to permanent stress events, such as metal contamination, by either selective growth (generalists) or introduction of metal-resistant species [52]. This natural process of selection will reserve species with the ability to adapt and survive, and eliminate sensitive species [53].

Distance-based redundancy analysis revealed that BCC was significantly affected by pH and heavy metal levels (cobalt). In addition, Spearman correlations indicated strong positive associations between pH with *Alpha*- and *Betaproteobacteria* and *Actinobacteria*. The relatively strong relationship to pH is not an unexpected finding. Previous studies have demonstrated that pH is an important environmental factor that influences and shapes community composition over a spatial gradient [54–56]. In fact, several studies have reported that pH was linearly correlated with the relative abundance of the main phyla [57–59].

Variation partitioning analysis in this study showed that mining was the main contributor to BCC variation, implicating the devastating impacts of gold mining not only on the water quality in the WFS, but also the composition and structure of microbial communities. The large proportion of unexplained variation by the four components is comparable to other comprehensive biogeographic studies [60, 61]. The result may also be attributed to additional factors not measured in this study, such as total heavy metal concentrations in sediments, organic nutrients, or other biotic factors. Moreover, sampling effects and ecological stochasticity (evolutionary, demographic, compositional or neutral processes) may further contribute to the unexplained proportion of microbial community variation [62, 63]

Cobalt concentration was another important source of variation in this study, which has been implicated in a limited number of studies on aquatic bacterial communities [45, 64, 65]. In addition, several individual taxa displayed positive associations with arsenic, chromium, nickel and uranium. These positive correlations may imply that specialized species are required in terms of biogeochemical functions in the WFS. Of particular importance are the genera *Aeromonas*, *Pseudomonas*, *Rhodoferax*, *Sphingopyxis*, *Flavobacterium*, *Hydrogenophaga* and *Dechloromonas*, which were observed in high abundance. These genera have been reported to exhibit a high degree of metal tolerance or reducing/oxidizing capability, in particular cobalt, arsenic, iron, chromium and mercury [66–69]. Moreover, these genera have been isolated from extreme environments such as metal-contaminated freshwaters and soil [70–72]. Given that our findings are based on phylogenetic and taxonomic classification of bacterial communities and that whole transcriptome changes across contamination loads were not measured in this study, our results do not reflect information on metal-resistant genes or any processes involved in metal oxidation/reduction. Future metagenomics research is required to determine metal-resistance genes in species to ascertain for the observed influence of cobalt on BCC.

Relatively little functional differences between upstream and downstream bacterial communities were inferred from PICRUSt analysis. However, variation in specific functional traits was observed between sites 1 and 4–5, which differ greatly with respect to location and degree of urbanization. Previous studies of aquatic bacterial communities found that the degree of urbanization had significant impacts on both community composition and functionality [73, 74]. These studies identified a link between specific taxa present and the community’s ability to utilize various carbon and nitrogen sources, suggesting functional variation may be attributed to both community membership and the influence of various land cover types [41]. In our study, both upstream and downstream sites had significant positive linear relationships between OTU and functional gene dissimilarities. The high linear regression slope for the downstream sites suggested that communities had more distinct functional genes, and thus higher functionality, than in upstream sites. Bacterial assemblages in upstream sites shared more functions like core resources metabolism and degradation of xenobiotics, indicating functional gene redundancy. This may be indicative that intense anthropogenic impacts can reduce functional diversity [74, 75]

Network analysis was used to explore interactions among OTUs, as well as specific associations with environmental parameters to identify key drivers of BCC in the WFS. Our ecological network suggests that environmental filtering (species sorting by local environmental conditions) affects community assembly in the lower WFS. The dominant taxa/generalists were assembled to an extent by environmental factors, indicating stronger species-sorting processes during the assembly of common taxa with evenly-distributed abundances [76].

The complete network, as well as the modules (IV-VI and VIII) constructed around the environmental parameters, showed that several OTUs were directly positively related to most variables. These included OTUs assigned to metal-resistant organisms (*Fluviicola*, *Nitrospira*), nitrifying and denitrifying bacteria (*Sphingopyxis*), phosphate removers (*Lacibacter*), sulfur oxidizers and/or sulfate reducers (*Cytophagales*, *Flavobacteriales*, *Burkholderiales*), and taxa involved in the degradation of complex organic polymers (*Fluviicola*). The negative associations between taxa and heavy metals are most likely due to the associated toxicity of the high metal concentrations. While some metals (e.g., iron, copper, zinc) are essential for normal bacterial growth and reproduction, high concentrations can cause adverse effects on bacterial communities such as a decrease in microbial biomass [77], changes in diversity [78], shifts in the dominant species [79], modulation of enzyme activities and the introduction of metal-resistant genes [80]. Furthermore, high metal concentrations might lead to suppressed microbial activities and element cycling processes [81, 82].

Although bacterial OTUs had strong associations with the environmental parameters, relationships among microbes dominated the network. For example, in module I, *Bacteroidetes* were positively connected with phylotypes within the phylum itself, as well as other prominent taxa (*Cyanobacteria*, *Firmicutes* and *Proteobacteria*). Strong positive linkages were also found (module II and IV) between *Proteobacteria* and *Bacteroidetes*, and between phylotypes within each respective taxon. This may indicate that some OTUs respond similarly to specific environmental factors rather than interacting directly and outcompeting others [83], some associations may be the result of substrate interdependencies [14], or it may indicate possible cooperation between species to cope with stress conditions [84]. Previous studies on aquatic microbial communities have demonstrated that closely-related taxa have coherent temporal dynamics and share similar ecological niches [85–87]. Conversely, negative associations between and within keystone taxa existed (e.g., *Alpha*- to *Gammaproteobacteria*, *Bacteroidetes* to *Proteobacteria* and *Cyanobacteria*, and *Betaproteobacteria* to itself). These may be indicative of non-overlapping niches, competitive behaviours, differences in growth constraints among taxa, or unique roles within the ecosystem [86, 88].

In conclusion, our results offer vital evidence that anthropogenic land uses, in particular mining, negatively impacted the water quality in the lower WFS and caused taxonomic and functional differences between upstream and downstream bacterial communities. Assemblages from more upstream sites (more polluted) have significantly different features than those presented by downstream sites (less contaminated). In terms of potential function, anthropogenic influenced upstream sites had higher relative abundances of genes encoding for core resource metabolism (carbohydrates, amino acids and lipids) and xenobiotic biodegradation compared to downstream sites that displayed greater functionality. The data recovered agreed with the expected assemblage of organisms thriving in freshwaters, metal-rich and sewage contaminated environments. Furthermore, our results indicated that pH and heavy metals were the major environmental parameters to significantly impact BCC and selective taxa in the lower WFS. The co-occurrence patterns presented here provide an initial glimpse of potential trait associations and mechanisms that drive community structure within this ecosystem. Overall, our results suggest that bacterial associations are more consistent with environmental filtering. Finally, a number of potential limitations and proposed furtherance need to be considered. (i) This study only measured the soluble fraction of heavy metals in the water column. Although informative, assessing heavy metal concentrations in sediments would reflect more accurately the pollution status of the WFS. Sediments are important sinks for various pollutants and metals, or can act as a non-point source to release pollutants and sediment-bound metals back to the overlying water column [89]. (ii) The time-series was too short to observe temporal changes in communities. Future studies should focus on longer time-series or seasonal-scale sampling to predict the dynamic patterns in BCC. (iii) The absence of functional data (metagenomics & metatranscriptomics) allowed us to only characterize BCC in terms of their taxonomy and phylogeny. Additional work on the WFS should characterize the metagenome- and metatranscriptome to elucidate the active metabolic pathways associated to this particular environment. (iv) A more complete physico-chemical data set is recommended since many parameters (e.g., DOC and biotic variables) known to impact BCC have not been measured in this study. (v) Co-occurrence patterns incorporating both generalist and specialists could be noteworthy as much of the unexplained variation in BCC could be attributed to specialists. It could also be possible that specialists were to a greater extent affected by environmental parameters not measured in this study. Such an extensive study may create a more comprehensive representation of the impacts of anthropogenic disturbances on water quality and bacterial communities in the WFS.

## Supporting information

S1 Fig Rarefaction curves for all sequences at all sampling locations estimating the number of bacterial OTUs at the 97% identity level. (TIF)

S2 Fig **Stacked column bar graph representing the predicted metabolic attributes between upstream and downstream sites against the KEGG database implemented in PICRUSt at (A) tier level-1 and (B) tier level-2.** The mean Nearest Sequenced Taxon Index (NSTI) value for all samples was 0.188 ± 0.000125 s.d.
(TIF)

S3 Fig Heat map and cluster analysis of bacterial core 97% identity OTUs between the different sampling locations. Samples were grouped using hierarchical clustering (complete linkage) based on the Bray–Curtis distance matrix calculated from the relative abundances (in percent) of the OTUs. The colour code goes from blue (not detected) to yellow (low abundance) to orange (medium abundance) to red (high abundance) on a logarithmic scale to improve visualization between low and medium abundance.
(TIF)

S4 Fig Network analysis of bacterial core OTUs (i.e. OTUs with relative abundance >0.2%) and environmental parameters. Co-occurrence patterns were based on significant (*P*<0.05) Spearman correlations with ρ ≥ ±0.6 showing the entire network structured according to site (upstream, downstream and/or both). Each node (circle) in the network represents a unique OTU and the size is proportional to node degree. Each edge (connection) represents a strong and significant correlation (*P*<0.05), while the colour relates to the type of interaction: positive (grey solid lines) or negative (red dashed lines). Environmental parameters are presented by purple rectangles.
(TIF)

S1 Table Summary of the sampling area, anthropogenic activities performed in the lower WFS and associated contaminants. (PDF)

S2 Table Recommended Target Water Quality Range (TWQR) for the lower Wonderfonteinspruit. (PDF)
