# Supplementary figures and images for: An integrated insight into the response of bacterial communities to anthropogenic contaminants in a river: A case study of the Wonderfonteinspruit catchment area, South Africa
Source: PLoS One. 2019 May 21;14(5):e0216758. doi: 10.1371/journal.pone.0216758 (PMC6528982; doi:10.1371/journal.pone.0216758)

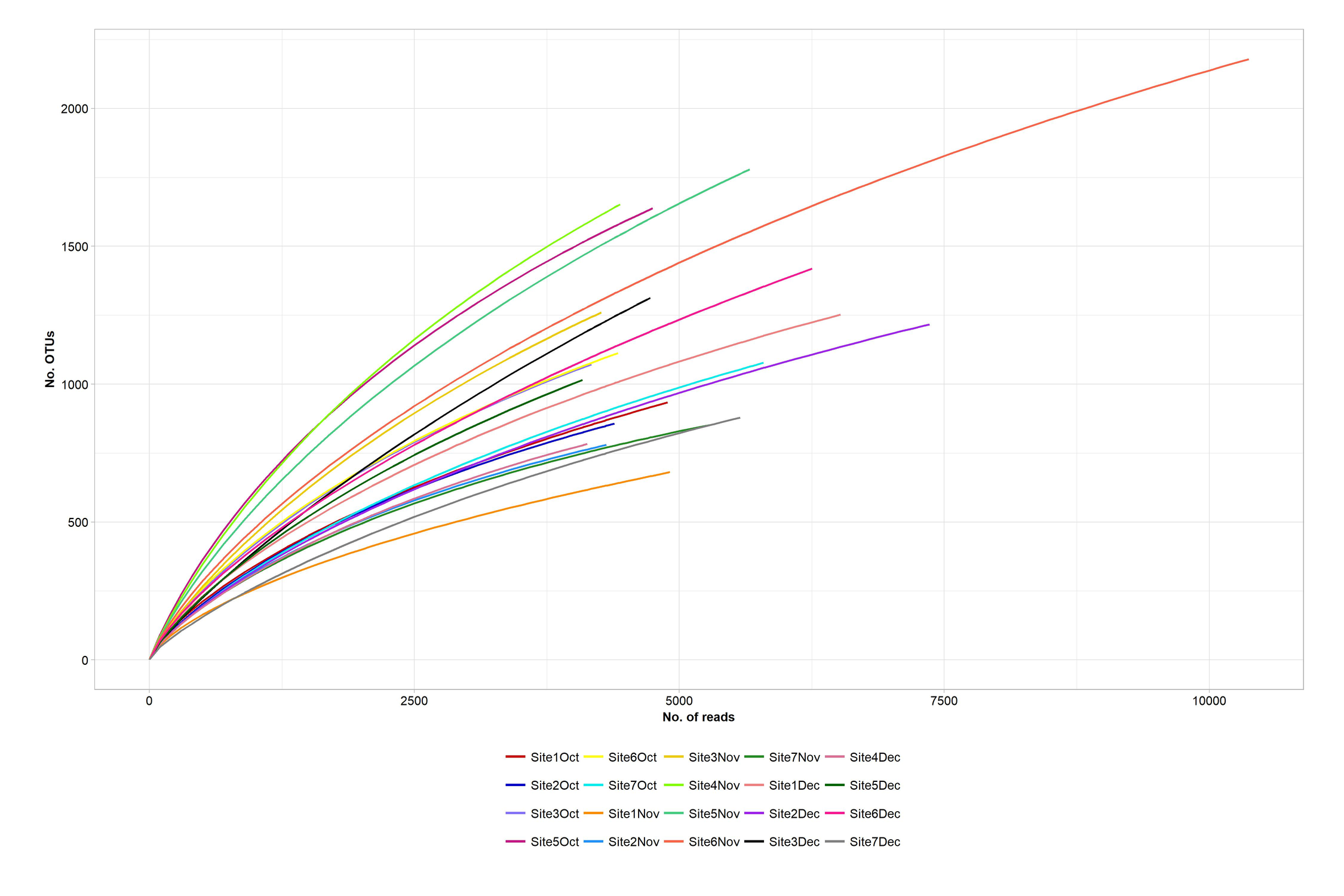

Supplement: S1 Fig — (TIF) [file pone.0216758.s001.tif]

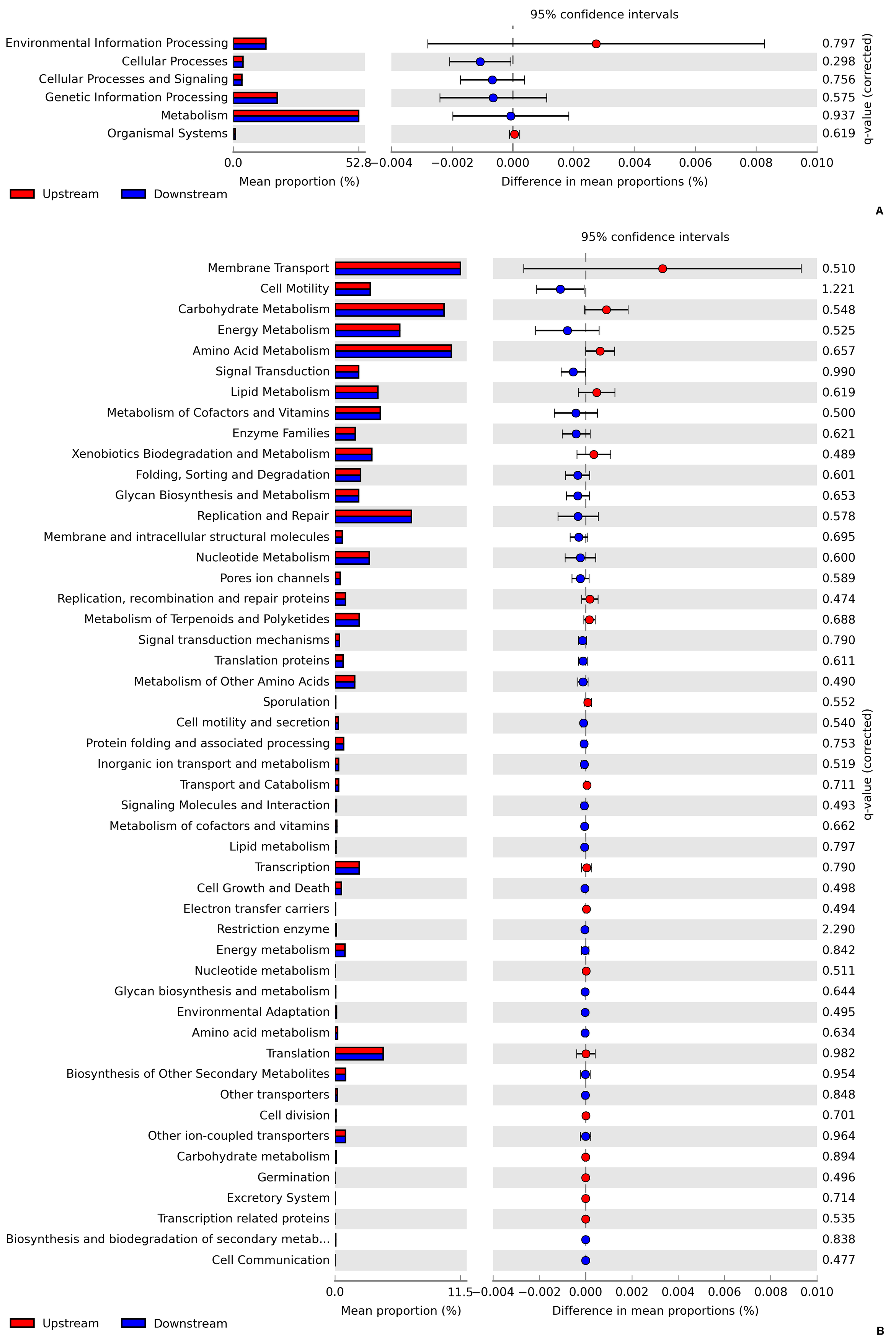

Supplement: S2 Fig — Stacked column bar graph representing the predicted metabolic attributes between upstream and downstream sites against the KEGG database implemented in PICRUSt at (A) tier level-1 and (B) tier level-2. The mean Nearest Sequenced Taxon Index (NSTI) value for all samples was 0.188 ± 0.000125 s.d. (TIF) [file pone.0216758.s002.tif]

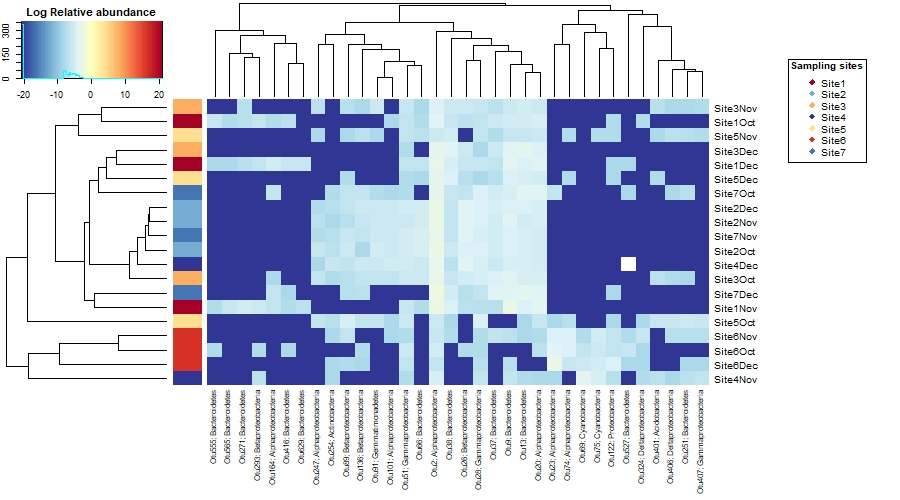

Supplement: S3 Fig — Samples were grouped using hierarchical clustering (complete linkage) based on the Bray–Curtis distance matrix calculated from the relative abundances (in percent) of the OTUs. The colour code goes from blue (not detected) to yellow (low abundance) to orange (medium abundance) to red (high abundance) on a logarithmic scale to improve visualization between low and medium abundance. (TIF) [file pone.0216758.s003.tif]

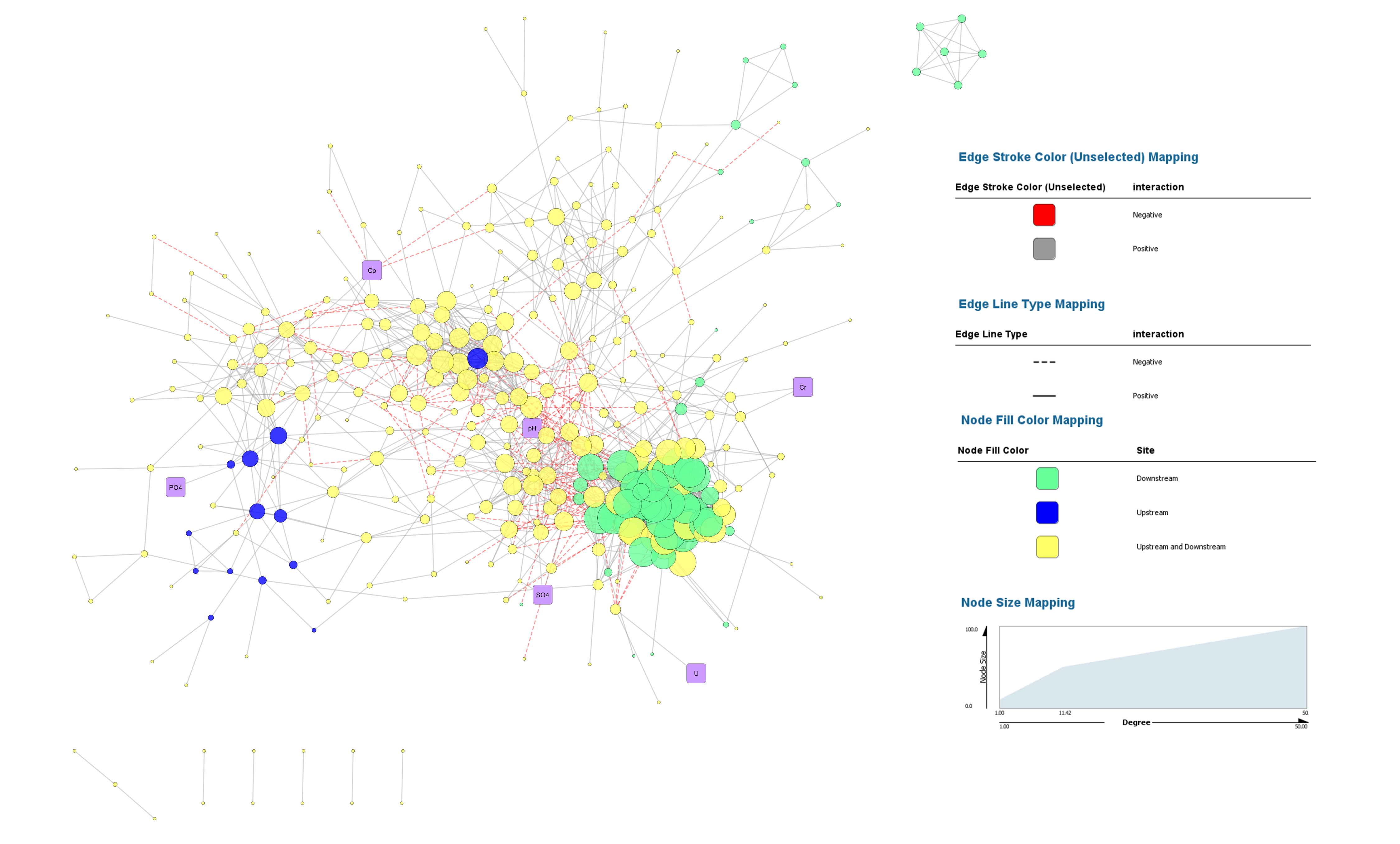

Supplement: S4 Fig — Co-occurrence patterns were based on significant (P<0.05) Spearman correlations with ρ ≥ ±0.6 showing the entire network structured according to site (upstream, downstream and/or both). Each node (circle) in the network represents a unique OTU and the size is proportional to node degree. Each edge (connection) represents a strong and significant correlation (P<0.05), while the colour relates to the type of interaction: positive (grey solid lines) or negative (red dashed lines). Environmental parameters are presented by purple rectangles. (TIF) [file pone.0216758.s004.tif]
