# Supplementary material for: An integrated insight into the response of bacterial communities to anthropogenic contaminants in a river: A case study of the Wonderfonteinspruit catchment area, South Africa
Source: PLoS One. 2019 May 21;14(5):e0216758. doi: 10.1371/journal.pone.0216758 (PMC6528982; doi:10.1371/journal.pone.0216758)
Supplement: S1 Table — (PDF) [file pone.0216758.s005.pdf]

S1 Table. Summary of the sampling area, anthropogenic activities performed in the lower WFS and associated contaminants.

Site	Location	Coordinates	Activity	Type of pollution	Contaminants
1	Carletonville	-26.31583333, 27.38247222	– Gold mining<br>– Urban<br>– Informal settlements	– Point discharge of mine effluents (fissure & process water) via pipelines & canals<br>– Point discharge of wastewater from municipal sewage works upstream of Carletonville<br>– Diffuse sources of pollution (e.g. storm runoff) from informal settlements	– Heavy metals (uranium, chromium, cobalt, nickel, copper, arsenic, cadmium, zinc)<br>– Microbial pathogens<br>– Salts<br>– Nutrients<br>– Phosphorus<br>– Nitrogen, Nitrate, Ammonium
2	Welverdiend	-26.3671944, 27.2705833	– Gold mining<br>– Urban<br>– Informal settlements	– Point discharge of mine effluents (fissure & process water) via pipelines & canals<br>– Point discharge of wastewater from Welverdiend municipal sewage works<br>– Diffuse sources of pollution (e.g. storm runoff) from informal settlements	– Heavy metals (uranium, chromium, cobalt, nickel, copper, arsenic, cadmium, zinc)<br>– Microbial pathogens<br>– Salts<br>– Nutrients<br>– Phosphorus<br>– Nitrogen, Nitrate, Ammonium
3	C2H069	-26.3700278, 27.2493889	Gold mining	– Point discharge of mine effluents (fissure & process water) via pipelines & canals<br>– Point discharge of wastewater from Welverdiend municipal	– Heavy metals (uranium, chromium, cobalt, nickel, copper, arsenic, cadmium, zinc)<br>– Microbial pathogens

				sewage works	– Nutrients<br>– Phosphorus<br>– Nitrogen, Nitrate, Ammonium
4	Turffontein dolomitic eye	-26.4095, 27.177417	Agriculture	– Diffuse pollution via seepage of fertilizers from irrigated crop areas<br>– Point & diffuse uranium pollution of groundwater sources by re-occurring short-term events (e.g. rain storms, floods, discharge events, accidental mine effluent spills)	– Heavy metals (uranium, chromium, cobalt, nickel, copper, arsenic, cadmium, zinc)<br>– Microbial pathogens<br>– Salts<br>– Nutrients<br>– Phosphorus<br>– Nitrate, Ammonium, Nitrogen oxides<br>– Pesticides
5	Muiskraal	-26.4364722, 27.1514167	Agriculture	– Diffuse pollution via seepage of fertilizers from irrigated crop areas<br>– Point & diffuse uranium pollution of groundwater sources by re-occurring short-term events (e.g. rain storms, floods, discharge events, accidental mine effluent spills)	– Heavy metals (uranium, chromium, cobalt, nickel, copper, arsenic, cadmium, zinc)<br>– Microbial pathogens<br>– Salts<br>– Nutrients<br>– Phosphorus<br>– Nitrate, Ammonium, Nitrogen oxides<br>– Pesticides
6	Gerhard Minnebron	-26.4798056,	Agriculture	– Diffuse pollution via seepage of	– Heavy metals (uranium,

	dolomitic eye	27.1516111		fertilizers from irrigated crop areas<br>– Point & diffuse uranium pollution of groundwater sources by re-occurring short-term events (e.g. rain storms, floods, discharge events, accidental mine effluent spills)	chromium, cobalt, nickel, copper, arsenic, cadmium, zinc)<br>– Microbial pathogens<br>– Salts<br>– Nutrients<br>– Phosphorus<br>– Nitrate, Ammonium, Nitrogen oxides<br>– Pesticides
7	Mooi River confluence	-26.514555, 27.1245278	Agriculture	– Diffuse pollution via seepage of fertilizers from irrigated crop areas	– Microbial pathogens<br>– Salts<br>– Nutrients<br>– Phosphorus<br>– Nitrate, Ammonium, Nitrogen oxides<br>– Pesticides
