# Supplementary material for: An integrated insight into the response of bacterial communities to anthropogenic contaminants in a river: A case study of the Wonderfonteinspruit catchment area, South Africa
Source: PLoS One. 2019 May 21;14(5):e0216758. doi: 10.1371/journal.pone.0216758 (PMC6528982; doi:10.1371/journal.pone.0216758)
Supplement: S2 Table — (PDF) [file pone.0216758.s006.pdf]

S2 Table. Recommended Target Water Quality Range (TWQR) for the lower Wonderfonteinspruit.

Variable	Unit	Domestic use	Irrigation	Livestock watering
pH		6.0 – 9.0	6.5 – 8.4	NA
EC	mS/m	0 – 70	0 – 40	NA
Nitrate (NO_3^-)	mg/L	0 – 6	0 – 5	0 – 100
Sulphate (SO_4^{2-})	mg/L	0 – 200	NA	0 - 1 000
Chloride (Cl^-)	mg/L	0 – 100	0 – 1.0	0 – 1500 (a)<br>0 – 3000 (b)
As	ppm	0 – 0.01	0 – 0.1	0 – 1
Cd	ppm	0 – 5	0 – 10	0 – 10
Co	ppm	NA	0 – 0.05	0 – 1
Cr	ppm	0 – 0.05	0 – 0.1	0 – 1
Cu	ppm	0 – 1	0 - 0.2	0 - 0.5 (c)<br>0 - 1 (d)<br>0 - 5 (e)
Fe	ppm	0 – 0.1	0 – 5	0 – 10
Hg	ppm	0 - 0.001	NA	0 - 1.0
Mn	ppm	0 – 0.05	0 - 0.02	0 - 10
Ni	ppm	NA	0 - 0.20	0 - 1
Pb	ppm	0 – 0.01	0 - 0.2	0 - 0.1 (f)<br>0 - 0.5 (g)
Se	ppm	0 - 0.02	0 - 0.02	0 - 50
U	ppm	0 – 0.070	NA	NA
Zn	ppm	0 – 3	0 - 1	0 - 20

(a) Monogastrics & Poultry

3 (b) Other Livestock

4 (c) Sheep & pre-weaned calves

5 (d) Cattle

6 (e) Horses, pigs & poultry

7 (f) All other livestock

8 (g) Pigs
